# Amyotrophic lateral sclerosis-associated mutant SOD1 inhibits anterograde axonal transport of mitochondria by reducing Miro1 levels

**DOI:** 10.1093/hmg/ddx348

**Published:** 2017-09-14

**Authors:** Annekathrin Moller, Claudia S Bauer, Rebecca N Cohen, Christopher P Webster, Kurt J De Vos

**Affiliations:** 1 Sheffield Institute for Translational Neuroscience (SITraN), Department of Neuroscience, University of Sheffield, Sheffield S10 2HQ, UK

## Abstract

Defective axonal transport is an early neuropathological feature of amyotrophic lateral sclerosis (ALS). We have previously shown that ALS-associated mutations in Cu/Zn superoxide dismutase 1 (SOD1) impair axonal transport of mitochondria in motor neurons isolated from SOD1 G93A transgenic mice and in ALS mutant SOD1 transfected cortical neurons, but the underlying mechanisms remained unresolved. The outer mitochondrial membrane protein mitochondrial Rho GTPase 1 (Miro1) is a master regulator of mitochondrial axonal transport in response to cytosolic calcium (Ca^2+^) levels ([Ca^2+^]_c_) and mitochondrial damage. Ca^2+^ binding to Miro1 halts mitochondrial transport by modifying its interaction with kinesin-1 whereas mitochondrial damage induces Phosphatase and Tensin Homolog (PTEN)-induced Putative Kinase 1 (PINK1) and Parkin-dependent degradation of Miro1 and consequently stops transport. To identify the mechanism underlying impaired axonal transport of mitochondria in mutant SOD1-related ALS we investigated [Ca^2+^]_c_ and Miro1 levels in ALS mutant SOD1 expressing neurons. We found that expression of ALS mutant SOD1 reduced the level of endogenous Miro1 but did not affect [Ca^2+^]_c_. ALS mutant SOD1 induced reductions in Miro1 levels were Parkin dependent. Moreover, both overexpression of Miro1 and ablation of PINK1 rescued the mitochondrial axonal transport deficit in ALS mutant SOD1-expressing cortical and motor neurons. Together these results provide evidence that ALS mutant SOD1 inhibits axonal transport of mitochondria by inducing PINK1/Parkin-dependent Miro1 degradation.

## Introduction

Amyotrophic lateral sclerosis (ALS) is an adult onset neurodegenerative disorder, which is characterized by the selective death of motor neurons in the spinal cord, motor cortex and brain stem (1). Approximately 10% of all ALS cases are inherited and mutations in a number of genes have been associated with ALS, including *superoxide dismutase 1* (*SOD1*), *TAR DNA binding protein* (*TARDBP*; TDP-43), *Fused in sarcoma* (*FUS*), and *C9orf72* (2). Approximately 185 different mutations in the enzyme Cu/Zn superoxide dismutase-1 (SOD1) have been associated with ALS (3) and it is generally accepted that these mutations cause ALS by toxic-gain-of-function mechanisms. Several toxic mechanisms have been proposed including, among others, excitotoxicity, neuroinflammation, loss of protein homeostasis, mitochondrial dysfunction, and defective axonal transport (4–7).

Neuronal function, integrity and survival are largely dependent on the correct distribution of proteins and organelles to their designated destinations. The architecture of neurons renders them particularly vulnerable to disruptions of transport processes and numerous lines of evidence suggest that defective axonal transport contributes to human neurodegenerative diseases including ALS (8). We and others have shown that impaired axonal transport is a very early event in both *in vitro* and *in vivo* models of ALS (5). In case of SOD1-related ALS we have shown that ALS mutant SOD1 selectively reduces anterograde transport of mitochondria in primary cortical and motor neuron cultures (9). Subsequent time-lapse recordings in single axons in the intact sciatic nerve of presymptomatic SOD1 G93A transgenic mice and rats revealed deficits in both anterograde and retrograde axonal transport of mitochondria *in vivo* (10–12).

Molecular motors transport mitochondria bi-directionally along axonal microtubules in response to physiological stimuli and energy requirements. Anterograde axonal transport of mitochondria is mediated by kinesin-1 whereas retrograde transport is mediated by cytoplasmic dynein. Both kinesin-1 and cytoplasmic dynein connect to mitochondria via mitochondrial Rho GTPase 1 (Miro1) and the adaptor proteins Trafficking Kinesin Protein (TRAK) 1 and 2 (13–22). Miro1 is a key regulator of axonal mitochondrial transport in response to calcium (Ca^2+^) and mitochondrial damage (16,23–26). Binding of Ca^2+^ to the Miro1 EF-hand motifs modulates its interaction with kinesin-1 such that the kinesin-1 motor domain directly binds to Miro1 and is thereby blocked from binding to microtubules, or, alternatively, kinesin-1 is released from mitochondria (24,25). Mitochondrial damage leads to phosphorylation of Miro1 by Phosphatase and Tensin Homolog (PTEN)-induced Putative Kinase 1 (PINK1) which targets Miro1 for Parkin-dependent proteasomal degradation and consequently causes an irreversible detachment of molecular motors from the mitochondrial surface (23,26,27).

Here we investigated the mechanism underlying impaired axonal transport of mitochondria in SOD1-related ALS. We report that ALS mutant SOD1 reduces anterograde mitochondrial transport by inducing PINK1/Parkin-dependent degradation of Miro1.

## Results

### ALS mutant SOD1 impairs axonal transport of mitochondria after lentiviral delivery in primary rat motor neurons

**Figure 1.**
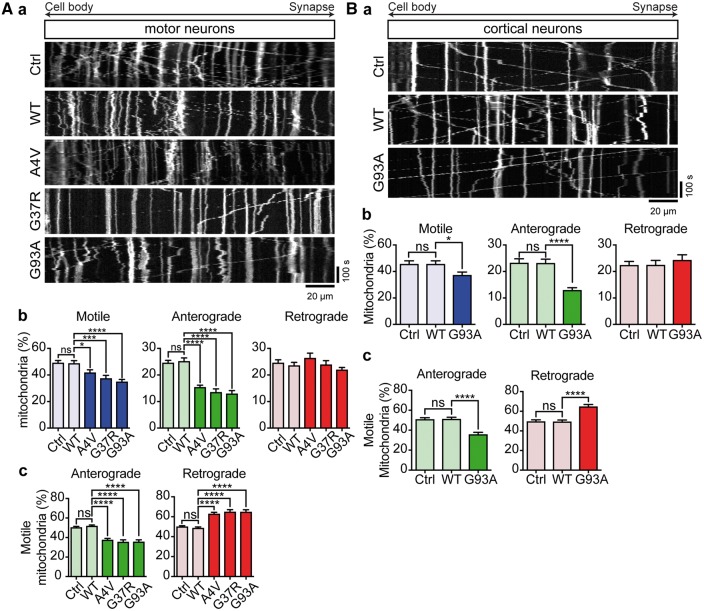
ALS mutant SOD1 impairs axonal transport of mitochondria. Axonal transport of mitochondria was analyzed in motor neurons (**A**) and cortical neurons (**B**). (Aa, Ba) Kymographs show transport of mitochondria in rat motor neurons and cortical neurons expressing EGFP (Ctrl), EGFP-SOD1 WT, A4V, G37R or G93A. (Ab, c; Bb, c) Quantitative analysis of mitochondrial transport shows that expression of ALS mutant SOD1 significantly impairs overall motility of mitochondria (Ab, Bb—Motile) because of a selective block of anterograde (Ab, Bb—Anterograde), but not retrograde (Ab, Bb—Retrograde) transport. As a consequence, SOD1 G93A disturbed the balance of transport to promote net retrograde movement (Ac, Bc). Results are shown as mean ± SEM, statistical significance was determined by one-way ANOVA followed by Fisher’s LSD test, ns, not significant, * *P* < 0.05, **** *P* < 0.0001, N (cortical neurons): Ctrl: 24, WT: 21, G93A: 24 from 5 experiments; N (motor neurons) = Ctrl: 16, WT: 20, A4V: 20, G37R: 21, G93A: 21 from 3 experiments.

We have shown previously that ALS mutant SOD1 disrupts axonal transport of mitochondria in transfected primary embryonic rat cortical neurons and in embryonic SOD1 G93A transgenic mouse motor neurons (9). To test if this defect could be recapitulated by expressing ALS mutant SOD1 in primary cultures of embryonic rat motor neurons we delivered EGFP, EGFP-SOD1 wild type (WT), A4V, G37R or G93A to DIV3 rat motor neurons by lentiviral transduction and analyzed axonal transport of MitoTracker Red CMXRos-labelled mitochondria from time-lapse recordings and corresponding kymographs as described by us before (Fig. 1A, a) (9,28,29). As a positive control, we quantified axonal transport in rat cortical neurons co-transfected with EGFP, EGFP-SOD1 WT or G93A and DsRed2-mito (Fig. 1B, a) (9).

In EGFP and EGFP-SOD1 WT transduced motor neurons approximately 50% of mitochondria were motile and anterograde and retrograde transport was balanced (Fig. 1A, b and c). Lentiviral delivery of EGFP-SOD1 A4V, G37R or G93A significantly reduced anterograde transport of mitochondria while retrograde transport remained unchanged (Fig. 1A, b); as a result, the balance of transport shifted toward net retrograde transport (Fig. 1A, c).

In EGFP and EGFP-SOD1 WT-expressing cortical neurons approximately 45% of mitochondria were motile, with near equal amounts of mitochondria moving in anterograde and retrograde directions (Fig. 1B, b). EGFP-SOD1 G93A caused a significant reduction in the number of anterograde mitochondria but did not affect the number of retrograde mitochondria (Fig. 1B, b). As a result, there was a significant shift toward net retrograde mitochondrial transport (Fig. 1B, c). Transfection of ALS mutant SOD1 did not affect axon length (Supplementary Material, Fig. S1).

Hence lentiviral delivery of ALS mutant SOD1 in embryonic rat motor neurons caused impairment of axonal transport of mitochondria similar to that reported in embryonic SOD1 G93A transgenic mouse motor neurons and transfected cortical neurons (9). Taken together these data also indicate that for the purpose of studying the effect of ALS mutant SOD1 on axonal transport of mitochondria, the method of delivery of SOD1, transfection or lentiviral transduction, and the neuronal cell type, cortical or motor neurons, are interchangeable.

### ALS mutant SOD1 does not affect cytosolic Ca^2+^ levels

We reported previously that expression of the ALS type-8 (ALS8)-associated vesicle-associated membrane protein-associated protein B (VAPB) mutant VAPBP56S in rat cortical neurons impairs anterograde transport of mitochondria similar to ALS mutant SOD1 (29). Mechanistically, overexpression of VAPBP56S increased cytosolic Ca^2+^ levels ([Ca^2+^]_c_), which caused a reduction in the amounts of tubulin but not kinesin-1 that were associated with Miro1 and as a consequence transport was arrested (29,30).

**Figure 2.**
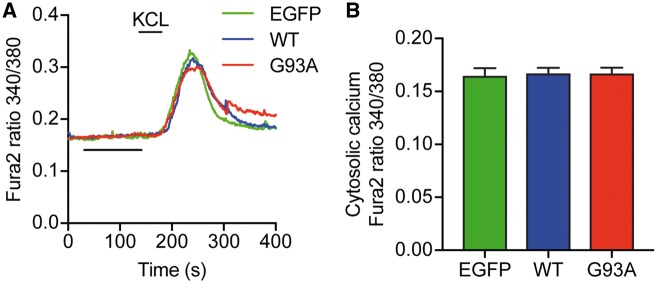
ALS mutant SOD1 G93A does not affect resting [Ca^2+^]_c_. Cortical neurons were transduced with EGFP, EGFP-SOD1 WT or G93A lentivirus and [Ca^2+^]_c_ determined by of Fura2 ratio imaging. To ensure that only viable neurons were taken into account, a transient Ca^2+^ influx was invoked by depolarization with 50 mM KCl. Average Ca^2+^ traces are shown in (**A**). Resting [Ca^2+^]_c_ was calculated as the average [Ca^2+^]_c_ between 50 and 150 s of recording and values for individual cells averaged to generate the bar graphs in (**B**). SOD1 G93A did not change resting [Ca^2+^]_c_ in comparison to EGFP control or SOD1 WT-expressing neurons. Results are shown as mean ± SEM, statistical significance was determined by one-way ANOVA, N (cells) = EGFP: 32, WT: 36, G93A: 37 from 3 experiments.

To investigate whether elevated [Ca^2+^]_c_ also played a role in ALS mutant SOD1-induced defective axonal transport of mitochondria, we examined resting [Ca^2+^]_c_ in cortical neurons expressing EGFP, EGFP-SOD1 WT or G93A by Fura2 ratio imaging. To identify viable neurons we induced a transient Ca^2+^ influx by depolarization with 50 mM KCl; only viable neurons were included in the analyses of resting [Ca^2+^]_c_. There was no difference in resting [Ca^2+^]_c_ in EGFP, EGFP-SOD1 WT and EGFP-SOD1 G93A-expressing neurons, suggesting that the axonal transport defect in ALS mutant SOD1 expressing neurons is not driven by changes in [Ca^2+^]_c_ (Fig. 2; Supplementary Material, Fig. S2).

### ALS mutant SOD1 reduces Miro1 levels in a Parkin-dependent fashion

It is well established that ALS mutant SOD1 accumulates in mitochondria and causes mitochondrial damage (7), and mitochondrial dysfunction has been linked to Miro1 degradation and impairment of axonal transport of mitochondria (23). Furthermore, Miro1 levels have been shown to be decreased in the spinal cord of transgenic mice expressing ALS mutant SOD1 G93A (31). To investigate if reduced levels of Miro1 may be at the basis of the axonal transport defect in SOD1-related ALS we first explored if expression of ALS mutant SOD1 could affect Miro1 levels in HEK293 cells and primary cortical neuron cultures.

**Figure 3.**
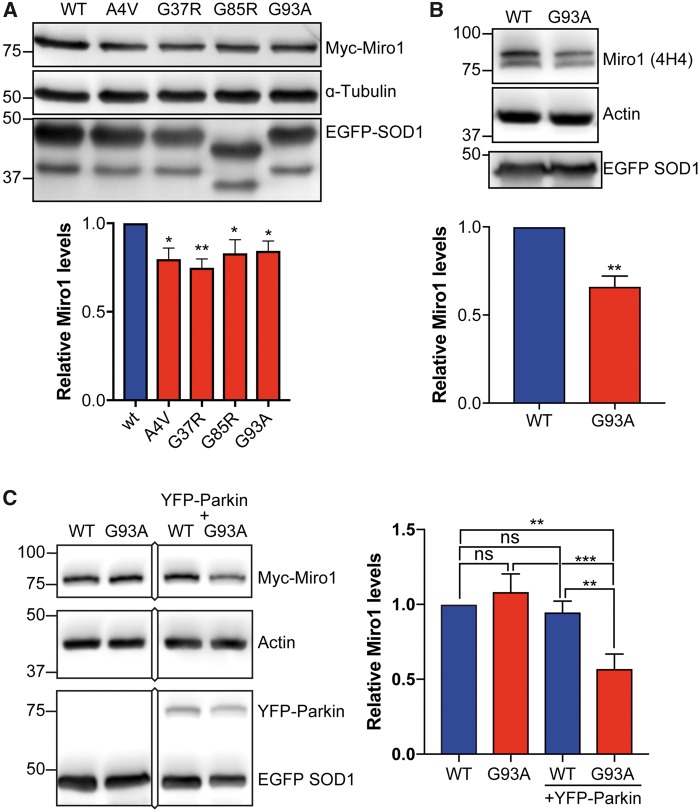
ALS mutant SOD1 reduces Miro1 expression levels via Parkin. (**A, B**) Western blot analysis of myc-Miro1 levels in HEK293 cells expressing EGFP-SOD1 WT, A4V, G93A, or G37R (A) or of endogenous Miro1 in cortical neurons expressing EGFP-SOD1 WT or G93A (B). ALS mutant SOD1 caused a decrease in Miro1 levels. Miro1 levels were corrected for loading using the α-tubulin or actin loading control and are shown relative to SOD1 WT. Expression of EGFP-SOD1 was verified on separate blots. (**C**) Western blot analysis of myc-Miro1 levels in HeLa cells expressing EGFP-SOD1 WT or G93A with or without co-expression of YFP-Parkin. ALS mutant SOD1 G93A-induced degradation of Miro1 was Parkin dependent. Results are shown as mean ± SEM, statistical significance was determined by (A) one-way ANOVA followed by Fisher’s LSD test, N= WT: 8, A4V: 7, G37R: 6, G85R: 8, G93A: 8, (B) unpaired t-test, N = 4, or (C) one-way ANOVA followed by Fisher’s LSD test, N = 6, ns not significant, * *P* < 0.05, ** *P* < 0.01, *** *P* < 0.001.

We co-transfected HEK293 cells with myc-tagged Miro1 (Myc-Miro1) and EGFP-SOD1 WT, A4V, G93A, G37R or G85R and quantified Myc-Miro1 levels on immunoblots. Compared to SOD1 WT all four ALS-associated SOD1 mutants caused a decrease in Myc-Miro1 levels of approximately 20% (Fig. 3A). We next determined if ALS mutant SOD1 affected the amount of endogenous Miro1 in rat cortical neurons transduced with EGFP-SOD1 WT or G93A. In these samples, endogenous Miro1 was readily detected on immunoblots as a doublet with a molecular weight of approximately 80 kDa as described before (32). Compared to SOD1 WT, ALS mutant SOD1 G93A significantly reduced the level of endogenous Miro1 in cortical neurons indicating that the ALS mutant SOD1-associated impairment of mitochondrial axonal transport may be due to reduced Miro1 levels (Fig. 3B).

To further investigate the involvement of the PINK1/Parkin pathway in ALS mutant SOD1-induced reductions in Miro1 levels we took advantage of HeLa cells that lack endogenous Parkin (33,34). We co-transfected HeLa cells with EGFP-SOD1 WT or ALS mutant SOD1 G93A and myc-Miro1 and determined Miro1 levels. ALS mutant SOD1 G93A did not affect Miro1 levels in native HeLa cells lacking Parkin (Fig. 3C). To confirm that this lack of effect of SOD1 G93A was indeed due to Parkin deficiency we restored Parkin expression by co-transfection of YFP-Parkin. It has been shown previously that expression of YFP-Parkin in HeLa cells restores the PINK1/Parkin pathway (35). In presence of YFP-Parkin ALS mutant SOD1 G93A caused a marked decrease in Miro1 compared to SOD1 WT (Fig. 3C).

Together these data indicate that ALS mutant SOD1 induces PINK1/Parkin-dependent degradation of Miro1.

### Expression of Miro1 rescues the effects of ALS mutant SOD1 on mitochondrial motility

To further investigate the role of Miro1 in the axonal transport defects observed in ALS mutant SOD1-expressing neurons we enquired if expressing wild type Miro1 or a Ca^2+^ insensitive mutant of Miro1 in which the EF hands were disrupted (Miro1^E208K/E328K^) could rescue the effect of ALS mutant SOD1 on mitochondrial transport. Expression of either wild type Miro1 or Miro1^E208K/E328K^ should rescue the effect of ALS mutant SOD1 on mitochondrial transport if the defect was caused by reduced levels of Miro1, but only Miro1^E208K/E328K^ should rescue if the defect was Ca^2+^ dependent. Indeed, we have previously shown that in agreement with a Ca^2+^ dependent mechanism, expression of Miro1^E208K/E328K^ but not wild type Miro1 rescued defective mitochondrial axonal transport in VAPBP56S-expressing cortical neurons (29).

**Figure 4.**
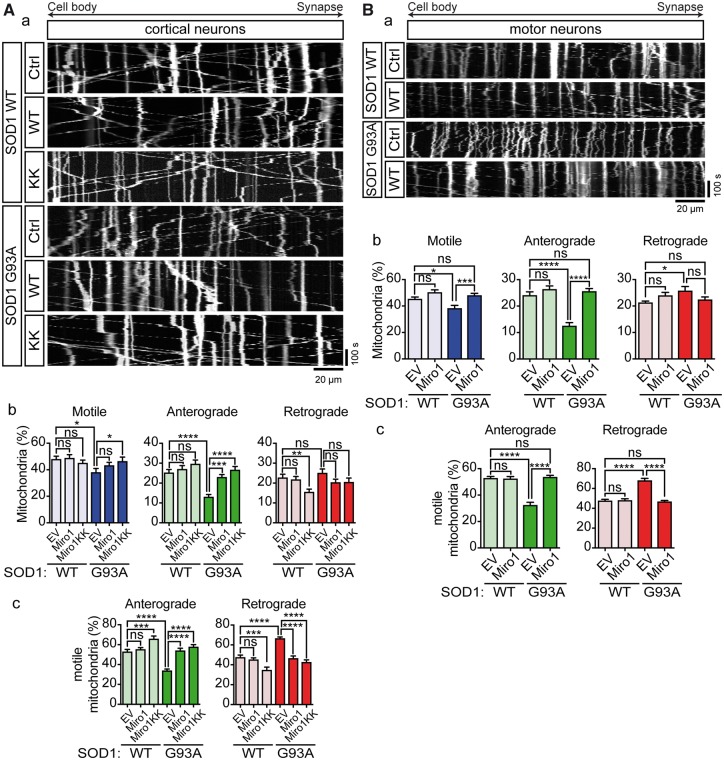
Expression of Miro1 rescues axonal transport of mitochondria in ALS mutant SOD1 expressing neurons. (**A**a, **B**a) Kymographs show transport of mitochondria in rat cortical neurons and motor neurons co-expressing EGFP-SOD1 WT or G93A with empty vector (Ctrl), myc-Miro1 (WT), or Myc-Miro1^E208K/E328K^ (KK). (Ab, c; Bb, c) Quantitative analysis of mitochondrial transport shows that expression of ALS mutant SOD1 significantly impairs overall motility of mitochondria (Ab, Bb—Motile) because of a selective block of anterograde (Ab, Bb—Anterograde), but not retrograde transport (Ab, Bb—Retrograde). As a consequence, SOD1 G93A disturbed the balance of transport to inhibit anterograde and promote retrograde movement (Ac, Bc). Co-expression of myc-Miro1 WT or KK, fully rescued impaired transport of mitochondria (Ab, Bb) and rebalanced anterograde and retrograde transport (Ac, Bc). Results are shown as mean ± SEM, statistical significance was determined by one-way ANOVA followed by Fisher’s LSD test, ns, not significant, * *P* < 0.05, *** *P* < 0.001, **** *P* < 0.0001, N (cortical neurons): SOD1 WT+Ctrl: 18, SOD1 WT+WT: 24, SOD1 WT+KK: 23, SOD1 G93A+Ctrl: 20, SOD1 G93A+WT: 24, SOD1 G93A+KK: 22 from 4 experiments; N (motor neurons) = WT+Ctrl: 15, SOD1 WT+WT: 16, SOD1 G93A+Ctrl: 19, SOD1 G93A+WT: 29 from 5 experiments.

We co-expressed Miro1 or Miro1^E208K/E328K^ with EGFP-SOD1 WT or G93A in rat primary cortical neurons and quantified transport of mitochondria. In SOD1 WT-expressing neurons Miro1^E208K/E328K^ but not Miro1 lowered the number of retrograde mitochondria (Fig. 4A, b). As a result, mitochondrial transport was skewed toward net anterograde transport in SOD1 WT + Miro1^E208K/E328K^ expressing neurons (Fig. 4A, c). In these neurons, the number of anterograde mitochondria was slightly elevated but not to a significant extent. These data are consistent with the previously proposed model in which binding of Ca^2+^ to the EF hands of Miro1 enables retrograde transport by inactivation of kinesin-1-mediated anterograde transport (24,25). Compared to SOD1 WT, transfection of SOD1 G93A alone again reduced anterograde transport of mitochondria and consequently shifted the balance of transport toward net retrograde transport (Fig. 4A, b and c). Both wild type Miro1 and Miro1^E208K/E328K^ fully rescued anterograde axonal transport (Fig. 4A, b) and restored the balance of transport in SOD1 G93A-expressing neurons (Fig. 4A, c).

The rescue by wild type Miro1 was further confirmed in motor neurons by quantification of axonal transport of mitochondria in rat motor neurons co-transduced with wild type Miro1 and EGFP-SOD1 WT or G93A (Fig. 4B). Expression of SOD1 G93A in absence of wild type Miro1 reduced anterograde axonal transport of mitochondria compared to SOD1 WT and, as was the case in cortical neurons, co-expression of wild type Miro1 fully rescued the effect of SOD1 G93A on mitochondrial transport (Fig. 4B, b and c). Together these data confirm that ALS mutant SOD1 does not act via Ca^2+^ but by reducing Miro1 levels to affect anterograde transport of mitochondria.

### Knockdown of PINK1 rescues the effects of ALS mutant SOD1 on mitochondrial motility

The above data strongly suggested that ALS mutant SOD1 impairs axonal transport of mitochondria by triggering PINK1/Parkin-dependent degradation of Miro1. To further verify this mechanism, we disrupted the PINK1/Parkin pathway by ablation of PINK1 expression using miRNA in cortical and motor neurons expressing EGFP-SOD1 WT or G93A and analyzed axonal transport of mitochondria. As control, we used a non-targeting control miRNA. The efficiency of the PINK1 miRNA-mediated knockdown was verified by qRT-PCR; on average 70% knockdown was achieved (Supplementary Material, Fig. S3).

**Figure 5.**
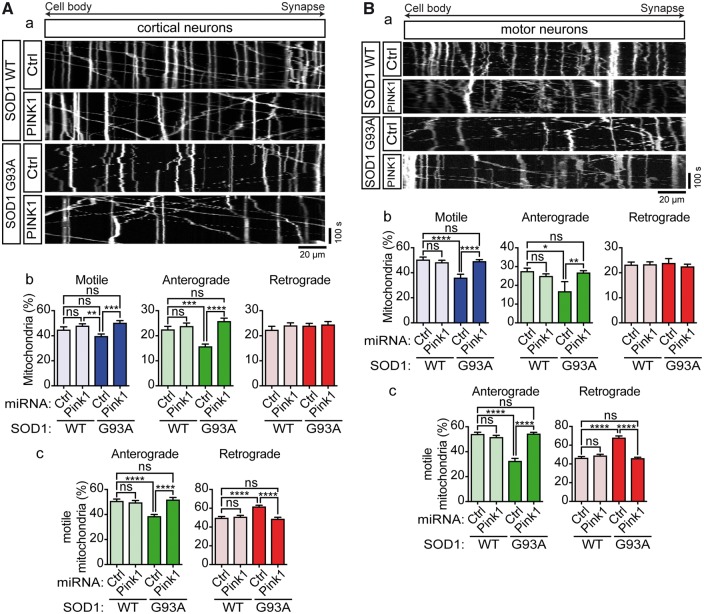
Knockdown of PINK1 rescues axonal transport of mitochondria in ALS mutant SOD1 expressing neurons. (**A**a, **B**a) Kymographs show transport of mitochondria in rat cortical neurons and motor neurons co-expressing EGFP-SOD1 WT or G93A with non-targeting control miRNA (Ctrl) or PINK1-targeting (PINK1) miRNA. (Ab, c; Bb, c) Quantitative analysis of mitochondrial transport shows that expression of ALS mutant SOD1 significantly impairs overall motility of mitochondria (Ab, Bb—Motile) because of a selective block of anterograde (Ab, Bb—Anterograde), but not retrograde transport (Ab, Bb—Retrograde). As a consequence, SOD1 G93A disturbed the balance of transport to inhibit anterograde and promote retrograde movement (Ac, Bc). Ablation of PINK1 expression fully rescued impaired transport of mitochondria (Ab, Bb) and rebalanced anterograde and retrograde transport (Ac, Bc). Results are shown as mean ± SEM, statistical significance was determined by one-way ANOVA followed by Fisher’s LSD test, ns, not significant, * *P* < 0.05, *** *P* < 0.001, **** *P* < 0.0001, N (cortical neurons): SOD1 WT+Ctrl: 25, SOD1 WT+PINK1: 32, SOD1 G93A+Ctrl: 39, SOD1 G93A+PINK1: 35 from 4 experiments; N (motor neurons) = SOD1 WT+Ctrl: 13, SOD1 WT+PINK1: 19, SOD1 G93A+Ctrl: 16, SOD1 G93A+PINK1: 27 from 4 experiments.

In non-targeting control miRNA + SOD1 WT expressing cortical and motor neurons approximately half of mitochondria were motile and these were near equally divided into anterograde and retrograde mitochondria (Fig. 5A and B, a and b). Control miRNA + SOD1 G93A-expressing cortical and motor neurons exhibited impaired anterograde transport of mitochondria (Fig. 5A and B, a and b) and this caused a shift toward net retrograde transport (Fig. 5A and B, c). Thus, control miRNA did not affect transport per se and did not alter SOD1 G93A-associated reductions in anterograde transport (compare with Fig. 1). In contrast, whereas PINK1 miRNA did not affect axonal transport of mitochondria in SOD1 WT-expressing cortical or motor neurons, it fully restored anterograde transport in SOD1 G93A-expressing neurons to control levels (Fig. 5A and B, b) and rebalanced transport (Fig. 5A and B, c). Thus, in agreement with a PINK1/Parkin-dependent reduction in Miro1 levels as the underlying cause of the ALS mutant SOD1-associated defect in mitochondrial trafficking, miRNA-mediated knockdown of PINK1 rescued mitochondrial axonal transport in SOD1 G93A-expressing cortical and motor neurons.

## Discussion

Defective axonal transport of mitochondria and other cargoes is one of the earliest neuropathological features observed in models of ALS which has led to the suggestion that it may play a causative role in disease (5). Mutations in essential axonal transport components such as cytoplasmic dynein, kinesin-1 and tubulin that have been shown to cause ALS and other motor neuron disorders, have reinforced this idea. We and others have shown that axonal transport of mitochondria is impaired in *in vitro* and *in vivo* models of mutant SOD1-related ALS but the underlying causes of defective transport remained unclear (9–12,36,37).

Here we show that impaired axonal transport of mitochondria in mutant SOD1-expressing neurons correlates with reduced levels of Miro1 (Fig. 3). Degradation of Miro1 has been shown to be an early event in PINK1/Parkin-dependent mitophagy. In mitophagy, mitochondrial damage causes stabilization of PINK1 on the outer mitochondrial membrane where PINK1 phosphorylates ubiquitin which in turn drives recruitment of Parkin, itself a PINK1 substrate, to the mitochondria (38). PINK1 also phosphorylates Miro1 which targets Miro1 for degradation in a Parkin-dependent manner (23). As a consequence, molecular motors are prevented from attaching to mitochondria and damaged mitochondria are immobilized. Consistent with PINK1-dependent reduction of Miro1 levels perturbing transport of mitochondria, expression of Miro1 and ablation of PINK1 rescued axonal transport in ALS mutant SOD1-expressing cortical and motor neurons (Figs 4 and 5). Furthermore, Miro1 degradation assays directly confirmed the Parkin dependent degradation of Miro1 (Fig. 3). It is well-established that ALS mutant SOD1 accumulates in mitochondria where it interacts with voltage-dependent anion channel 1 (VDAC1) and Bcl-2, and causes mitochondrial dysfunction (39–45). Thus, our data suggest that ALS mutant SOD1-induced mitochondrial damage activates the PINK1/Parkin pathway and as a result halts mitochondrial transport. In line with such a model it has been shown that Miro1-expression is decreased in the spinal cord of SOD1 G93A transgenic mice (31).

We previously reported that mutant VAPBP56S deregulates Ca^2+^ homeostasis leading to increased [Ca^2+^]_c_ that impairs mitochondrial transport by binding to the EF hand motifs of Miro1 (29). *In vitro* data on [Ca^2+^]_c_ in ALS mutant SOD1 expressing neurons is conflicting. SH-SY5Y cells stably expressing SOD1 G93A have been shown to have in increased [Ca^2+^]_c_. In contrast, both unchanged (46,47) and increased [Ca^2+^]_c_ (48) has been found in SOD1 G93A transgenic motor neuron cultures. In fact, a meta-analysis of 26 [Ca^2+^]_c_ values from 9 studies, found that half of the data points show elevated [Ca^2+^]_c_ and half show lower [Ca^2+^]_c_ in SOD1 G93A expressing neurons compared to SOD1 WT (49). We did not find any change in basal [Ca^2+^]_c_ upon ALS mutant SOD1 expression (Fig. 2 and Supplementary Material, Fig. S2) and consistent with a Ca^2+^-independent mechanism both wild type and Ca^2+^ insensitive mutant Miro1 were able to restore axonal transport of mitochondria in ALS mutant SOD1 expressing neurons (Fig. 4). Thus, two distinct genetic causes of ALS, mutant VAPB and mutant SOD1, that impair axonal transport of mitochondria converge on Miro1 to halt transport, albeit by different mechanisms.

Impaired axonal transport of mitochondria has also been observed in models of TDP-43, FUS, SIGMAR1 and C9orf72-related ALS (29,36,50–53). Furthermore, neuropathological studies of post-mortem ALS cases have revealed accumulations of mitochondria in the proximal segment of motor neurons and in axonal spheroids which are indicative of damaged axonal transport (54–56). It is not yet clear what the underlying causes of impaired axonal transport of mitochondria are in these cases, but mitochondrial damage and Ca^2+^ mishandling are well-documented in all of ALS (7,57). Both ALS mutant TDP-43 and FUS have been shown to accumulate in mitochondria and disrupt mitochondrial function (58,59). C9orf72 GGGGCC repeat expansion-associated glycine/arginine (GR) dipeptide repeat protein (DPR) has been shown to bind to mitochondrial ribosomes and compromise mitochondrial function (60). Mitochondria appear swollen and vacuolated in motor neurons of ALS patients (54). TDP-43, FUS, and SIGMAR1 have all been linked to Ca^2+^ mishandling, probably due to disruption of ER-mitochondria contact sites (53,61–65), and evidence suggests altered Ca^2+^ in the motor terminals of ALS patients (66). In agreement with mitochondrial damage-associated reductions in Miro1 causing axonal transport defects in non-SOD1 ALS, decreased levels of Miro1 have been reported in TDP-43 M337V transgenic mice and in ALS patients (31), and, in the case of FUS, a genetic interaction with PINK1/Parkin has been described in *Drosophila* (52). Thus, several ALS-associated insults may converge on axonal transport via Miro1 and trigger motor neuron demise.

Defective axonal transport of mitochondria leading to depletion of mitochondria from axons is one of the earliest phenomena observed in ALS models and has been proposed to play an important causal role in disease (5). However, increasing the motility of mitochondria in SOD1 G93A transgenic mice did not slow disease in these animals (67). Our data now suggest that mitochondrial damage is the upstream cause of defective axonal transport of mitochondria in mutant SOD1-associated ALS. Hence it may not be surprising that stimulating anterograde transport of damaged mitochondria did not alter the course of disease in SOD1 G93A transgenic mice. Indeed, our findings indicate that, at least in the case of ALS mutant SOD1, the relative increase in retrograde transport of mitochondria at the cost of anterograde transport represents increased clearance of damaged mitochondria via mitophagy. If this is also the case in non-SOD1 ALS is not yet clear but these results suggest that successful therapeutic strategies will probably need to target both mitochondrial dysfunction and transport simultaneously.

## Materials and Methods

### Plasmids

pDsRed2-Mito and pEGFPc2 were purchased from Clontech. pEGFPc2-SOD1 and pRK5-myc-Miro1^E208K/E328K^ expression constructs have been described before (9,29). pRK5-myc-Miro1 was a gift from P. Aspenström (Karolinska Institutet, Stockholm, Sweden) (17). pCMV-dR8.92 was a gift from Bob Weinberg via Addgene (plasmid #8455) (68). pMD2.G and pRSV-Rev were both gifts from Didier Trono via Addgene (plasmid #12253 and #12259) (69). YFP-Parkin was a gift from Alexander Whitworth (MRC Mitochondrial Biology Unit, Cambridge, UK).

A rat PINK1-targeting miRNA expression construct was designed using Invitrogen’s RNAi Designer (www.invitrogen.com/rnai) and cloned into the pcDNA6.2^TM^-GW/EmGFP-miR expression vector according to the manufacturer’s instructions (BLOCK-iT^TM^ Pol II miR RNAi expression vector kit, Invitrogen). Sequences for the top strands were 5′-TGC TGT GTC CTA TCA GAT AAT CCT CCG TTT TGG CCA CTG ACT GAC GGA GGA TTC TGA TAG GAC A (PINK1) and 5′-TGC TGA AAT GTA CTG CGC GTG GAG ACG TTT TGG CCA CTG ACT GAC GTC TCC ACG CAG TAC ATT T (negative control; provided with the kit). In order to exchange the EmGFP tag for a ECFP tag, ECFP was amplified using primers 5′-TTT AAA ACC ATG GTG AGC AAG GGC GAG GAG (fw) and 5′-TTT AAA CGA TCT TAC TTG TAC AGC TCG TCC ATG CC (rev) from pECFP (Clontech). After sub-cloning into pCR^®^-Blunt II-TOPO (Life Technologies), ECFP was cloned into pcDNA6.2^TM^-GW/EmGFP using DraI sites (Thermo Scientific).

For lentiviral transduction EmGFP or ECFP-miR-PINK1 and EGFP-SOD1 expression constructs, were subcloned in pLvos (70) using NheI and NotI sites yielding pLvos-EmGFP or ECFP-miR-PINK1, pLvos-EGFP-SOD1 WT, A4V, G37R and G93A. Virus production was as described before (70).

### Cell culture, plasmid transfection, lentiviral transduction

Cortical neurons from E18 rat embryos were isolated and cultured as described before (9,28,70). 375 000 cells were seeded onto 18 mm cover slips coated with poly-L-lysine. Cells were cultured for 5–7 d and then transfected with Lipofectamine LTX (Life Technologies; 0.5 µl/µg of DNA). Transduction with lentiviruses was performed on DIV5 using an MOI of 5.

Motor neurons from E15 rat embryos were isolated as described before (71) and cultured in Neurobasal^®^ Medium (Life Technologies) supplemented with 2% (v/v) B-27^®^ supplement (Life Technologies), 2% (v/v) heat-inactivated horse serum (Sigma), 2 mM GlutaMAX^TM^-I (Life Technologies), 25 µM L-glutamic acid (Sigma), 5 ng/ml brain-derived neurotrophic factor (BDNF), 10 ng/ml glial cell-derived neurotrophic factor (GDNF) and 10 ng/ml ciliary neurotrophic factor (CNTF; all neurotrophic factors were purchased from R&D Systems). 40 000 motor neurons were seeded onto 18 mm cover slips coated with poly-DL-ornithine (Sigma) and Laminin (Life Technologies). Transduction with lentiviruses was performed on DIV 0 using an MOI of 5.

HEK293 and Hela cells were cultured in high glucose Dulbecco’s Modified Eagle’s Medium (DMEM, Sigma) supplemented with 10% foetal calf serum (Sigma) and 1 mM sodium pyruvate (Sigma). HEK293 cells were transfected using Lipofectamine 2000 according to the manufacturer’s protocol (Life Technologies); Hela cells were transfected using polyethyleneimine (PEI, 1 mg/ml; 3:2 PEI:DNA; Polysciences Inc.).

### Antibodies

Mouse monoclonal anti-myc-tag antibody (9B11; 1:2000) was from Cell Signaling Technology. Mouse monoclonal anti-GFP antibody (JL8; 1:5000) was from Clontech. Rabbit polyclonal anti-RHOT1 antibody (1:1000), mouse monoclonal anti-RHOT1 (4H4; 1:1000), mouse monoclonal anti-actin (4C2; 1:5000) and mouse monoclonal anti-α-tubulin antibody (DM1A; 1:10 000) were from Sigma. Secondary antibodies used for immunoblotting were horseradish peroxidase-coupled goat anti-rabbit and rabbit anti-mouse IgG from Dako (1: 5000), and alkaline phosphatase-coupled goat anti-mouse and goat anti-rabbit IgG antibodies from Sigma (1:10 000).

### Miro1 assays

For Myc-Miro1 assays in HEK293 cells, cells were transfected with equal amounts of Myc-Miro1 and either EGFP-SOD1 WT, A4V, G37R, G85R or G93A. Cells were processed for immunoblot 24 h after transfection. In case of cortical neurons, the neurons were transduced at DIV5 and endogenous Miro1 levels were determined on immunoblot 10 days after lentiviral transduction. HeLa cells were transfected with Myc-Miro1, EGFP-SOD1 WT or G93A, and YFP-Parkin or pciNeo empty vector (ratio: 1:2:1). Myc-Miro1 levels were determined 48–72 h after transfection.

### SDS-PAGE and immunoblot

Samples were separated on poly-acrylamide gels and transferred to Protran nitrocellulose membranes (0.45 μm, GE Healthcare) using a Pierce G2 Fast Blotter (Thermo Scientific) or Bio-Rad TransBlot cell. Membranes were blocked for 1 h at room temperature in TBS containing 5% milk powder/0.1% Tween-20 (TBST-M) and probed with primary antibodies in TBST-M for either 1 h at room temperature or 16 h at 4 °C. After washing with TBS/0.1% Tween-20 (TBST), membranes were incubated with horseradish peroxidase (HRP)-conjugated or alkaline phosphatase (AP)-coupled, species-specific secondary antibodies. Following incubation with SuperSignal West Pico Chemiluminescence Substrate (Thermo Scientific) or AttoPhos^®^ AP Fluorescent Substrate (Promega) signals were detected on Hyperfilm ECL (GE Healthcare) or using a G: Box imager (Syngene). Films were scanned on a CanoScan LiDE 60 photo scanner and signals were quantified using the Fiji distribution (http://fiji.sc/) (72) of the ImageJ image processing software [National Institutes of Health (NIH), http://rsb.info.nih.gov/ij/] (73).

### Mitochondrial transport assays

For axonal transport assays in cortical neurons, 0.7 μg of pEGFPc2, or pEGFPc2-SOD1 were transfected together with 0.3 μg of pDsRed2-Mito or 0.2 μg of pDsRed2-Mito + 0.1 μg of either pRK5-myc-Miro1, Miro1^E208K/E328K^ or pcDNA6.2^TM^-GW/EmGFP-miR-PINK1. Motor neurons were transduced with lentiviruses on DIV0 using an MOI of 5.

Live microscopy of mitochondrial axonal transport was performed using an Axiovert200 microscope (Carl Zeiss) equipped with a Polychrome IV monochromator (Till Photonics), an EGFP/DsRed filter set (Chroma Technology Corp.), a 40× EC Plan-Neofluar 1.3 N.A. objective (Zeiss), a high speed HF110A emission filter wheel controlled by a ProScan III Controller (Prior Scientific) and a Hamamatsu C9100-12 EMCCD camera (Hamamatsu Photonics). The microscope setup was controlled using MicroManager 1.4.21 (74). 48 h post-transfection or 72 h post-transduction, coverslips were transferred to a heated observation chamber (SA-20LZ, Warner Instruments) mounted on the stage of the microscope. In order to visualize mitochondria, motor neurons were stained with MitoTracker^®^ Red CMXRos (66 nM, Life Technologies) beforehand. In addition to the heated chamber, an objective heater (IntraCell) was used to maintain the cells at 37 °C. Mitochondrial movements were recorded for 5 min with 3 s time-lapse interval in Neurobasal medium (Life Technologies) supplemented with 5 mM HEPES pH 7.0 and 2 mM GlutaMAX™-I. Axonal transport was analyzed using ImageJ as described previously (9,28,29). Briefly, the overall transport of mitochondria was quantified from kymographs by calculating the distance between the position of individual mitochondria at the start and end of time-lapse recordings and dividing by the time elapsed. This yielded an average transport velocity for each mitochondrion that includes anterograde and retrograde movements and stationary periods. Mitochondria were classified as motile when their velocity exceeded 0.1 µm/s or as stationary when their velocity was ≤0.1 µm/s. Mitochondria were classified as anterograde or retrograde according to their predominant direction of travel.

### Ca^2+^ imaging

Resting [Ca^2+^]_c_ levels in transfected cortical neurons were determined by Fura2 ratio imaging as described previously (29). First, cells were stained for 20 min at 37 °C with 5 μM Fura2-AM (Life Technologies) in external solution (ES; 145 mM NaCl, 2 mM KCl, 5 mM NaHCO_3_, 1 mM MgCl_2_, 2.5 mM CaCl_2_, 10 mM glucose, 10 mM HEPES pH 7.0). After destaining by incubation for another 20 min in ES only, Fura2 340 nm/Fura2 380 nm image pairs were recorded for 400 s with 1 s time-lapse interval using the same microscopy setup as described above, but using WinFluor V3.7.4 software (written by John Dempster, University of Strathclyde; http://spider.science.strath.ac.uk/sipbs/software_imaging.htm). Neurons were perfused continuously with ES (2 ml/min; Gilson Minipuls Evolution). To ensure that only viable neurons were taken into account, a transient Ca^2+^ influx was invoked by depolarization with 50 mM KCl for 1 min. Signals were recorded and analyzed using the WinFluor software.

### Quantitative real time PCR

RNA from cultured cells was isolated according to the manufacturer’s instructions using TRIzol^®^ Reagent (Life Technologies). After resuspension in nuclease-free water, 0.5–2 μg RNA were treated with DNase I [NEB; DNase I was inactivated by addition of 2.5 mM EDTA (final concentration) and incubation for 10 min at 75 °C] and reverse transcribed using SuperScript^®^ III Reverse Transcriptase and Oligo(dT) primers (Thermo Scientific). 100 ng of template was subjected to a quantitative real time PCR utilising Brilliant III Ultra-Fast 2× SYBR^®^ Green QPCR master mix (Agilent Technologies) or HOT Firepol EvaGreen PCR Mix Plus (Solis Biodyne) using an Mx3000p qPCR System (Agilent Technologies) or a BioRad CFX96 Real-Time System (C1000 Touch Thermal Cycler; BioRad). PINK1 primers were as follows: rnPINK1 fw: 5′-TGT CAG GAG ATC CAG GCA ATT, rnPINK1 rev: 5′-CTT CAT ACA CAG CGG CAT TGCA, GAPDH or RPL19 was used as endogenous control: rnGAPDH fw: 5′-TGA AGG GTG GGG CCA AAGG, rnGAPDH rev: 5′-GGT CAT GAG CCC TTC CAT GA, rnRPL19 fw: 5′- CTC GAT GCC GGA AGA ACA CC -3′, rnRPL19 rev: 5'- GAG CGT TGG CAG TAC CCT T -3′.

### Statistical analysis

Calculations and statistical analysis were performed using Excel (Microsoft Corporation) and GraphPad Prism software (GraphPad Software).

## Supplementary Material

Supplementary Material is available at *HMG* online.

## Supplementary Material

Supplementary Figure S1

Supplementary Figure S2

Supplementary Figure S3

## References

[1] Kiernan M.C., Vucic S., Cheah B.C., Turner M.R., Eisen A., Hardiman O., Burrell J.R., Zoing M.C. (2011) Amyotrophic lateral sclerosis. Lancet, 377, 942–955. 21296405 10.1016/S0140-6736(10)61156-7

[2] Renton A.E., Chio A., Traynor B.J. (2014) State of play in amyotrophic lateral sclerosis genetics. Nat. Neurosci., 17, 17–23. 24369373 10.1038/nn.3584 PMC4544832

[3] Abel O., Powell J.F., Andersen P.M., Al-Chalabi A. (2012) ALSoD: a user-friendly online bioinformatics tool for amyotrophic lateral sclerosis genetics. Hum. Mutat., 33, 1345–1351. 22753137 10.1002/humu.22157

[4] Webster C.P., Smith E.F., Shaw P.J., De Vos K.J. (2017) Protein homeostasis in amyotrophic lateral sclerosis: therapeutic opportunities. Front. Mol. Neurosci., 10, 123. 28512398 10.3389/fnmol.2017.00123 PMC5411428

[5] De Vos K.J., Hafezparast M. (2017) Neurobiology of axonal transport defects in motor neuron diseases: opportunities for translational research. Neurobiol. Dis., 105, 283–299. 28235672 10.1016/j.nbd.2017.02.004 PMC5536153

[6] Ferraiuolo L., Kirby J., Grierson A.J., Sendtner M., Shaw P.J. (2011) Molecular pathways of motor neuron injury in amyotrophic lateral sclerosis. Nat. Rev. Neurol., 7, 616–630. 22051914 10.1038/nrneurol.2011.152

[7] Smith E.F., Shaw P.J., De Vos K.J. (2017) The role of mitochondria in amyotrophic lateral sclerosis. Neurosci. Lett., pii: S0304-3940(17)30544-X. doi: 10.1016/j.neulet.2017.06.052. 10.1016/j.neulet.2017.06.052 28669745

[8] De Vos K.J., Grierson A.J., Ackerley S., Miller C.C. (2008) Role of axonal transport in neurodegenerative diseases. Annu. Rev. Neurosci., 31, 151–173. 18558852 10.1146/annurev.neuro.31.061307.090711

[9] De Vos K.J., Chapman A.L., Tennant M.E., Manser C., Tudor E.L., Lau K.F., Brownlees J., Ackerley S., Shaw P.J., McLoughlin D.M. (2007) Familial amyotrophic lateral sclerosis-linked SOD1 mutants perturb fast axonal transport to reduce axonal mitochondria content. Hum. Mol. Genet., 16, 2720–2728. 17725983 10.1093/hmg/ddm226 PMC4516806

[10] Marinkovic P., Reuter M.S., Brill M.S., Godinho L., Kerschensteiner M., Misgeld T. (2012) Axonal transport deficits and degeneration can evolve independently in mouse models of amyotrophic lateral sclerosis. Proc. Natl. Acad. Sci. USA, 109, 4296–4301. 22371592 10.1073/pnas.1200658109 PMC3306689

[11] Magrané J., Sahawneh M.A., Przedborski S., Estévez Á.G., Manfredi G. (2012) Mitochondrial dynamics and bioenergetic dysfunction is associated with synaptic alterations in mutant SOD1 motor neurons. J. Neurosci., 32, 229–242. 22219285 10.1523/JNEUROSCI.1233-11.2012 PMC3566782

[12] Bilsland L.G., Sahai E., Kelly G., Golding M., Greensmith L., Schiavo G. (2010) Deficits in axonal transport precede ALS symptoms in vivo. Proc. Natl. Acad. Sci. USA, 107, 20523–20528. 21059924 10.1073/pnas.1006869107 PMC2996651

[13] Morlino G., Barreiro O., Baixauli F., Robles-Valero J., González-Granado J.M., Villa-Bellosta R., Cuenca J., Sánchez-Sorzano C.O., Veiga E., Martín-Cófreces N.B. (2014) Miro-1 links mitochondria and microtubule Dynein motors to control lymphocyte migration and polarity. Mol. Cell. Biol., 34, 1412–1426. 24492963 10.1128/MCB.01177-13 PMC3993592

[14] Brickley K., Stephenson F.A. (2011) Trafficking kinesin protein (TRAK)-mediated transport of mitochondria in axons of hippocampal neurons. J. Biol. Chem., 286, 18079–18092. 21454691 10.1074/jbc.M111.236018 PMC3093881

[15] Russo G.J., Louie K., Wellington A., Macleod G.T., Hu F., Panchumarthi S., Zinsmaier K.E. (2009) Drosophila Miro is required for both anterograde and retrograde axonal mitochondrial transport. J. Neurosci., 29, 5443–5455. 19403812 10.1523/JNEUROSCI.5417-08.2009 PMC2693725

[16] Saotome M., Safiulina D., Szabadkai G., Das S., Fransson A., Aspenstrom P., Rizzuto R., Hajnóczky G. (2008) Bidirectional Ca2+-dependent control of mitochondrial dynamics by the Miro GTPase. Proc. Natl. Acad. Sci. USA, 105, 20728–20733. 19098100 10.1073/pnas.0808953105 PMC2634948

[17] Fransson S., Ruusala A., Aspenstrom P. (2006) The atypical Rho GTPases Miro-1 and Miro-2 have essential roles in mitochondrial trafficking. Biochem. Biophys. Res. Commun., 344, 500–510. 16630562 10.1016/j.bbrc.2006.03.163

[18] Glater E.E., Megeath L.J., Stowers R.S., Schwarz T.L. (2006) Axonal transport of mitochondria requires milton to recruit kinesin heavy chain and is light chain independent. J. Cell Biol., 173, 545–557. 16717129 10.1083/jcb.200601067 PMC2063864

[19] Guo X., Macleod G.T., Wellington A., Hu F., Panchumarthi S., Schoenfield M., Marin L., Charlton M.P., Atwood H.L., Zinsmaier K.E. (2005) The GTPase dMiro is required for axonal transport of mitochondria to Drosophila synapses. Neuron, 47, 379–393. 16055062 10.1016/j.neuron.2005.06.027

[20] Brickley K., Smith M.J., Beck M., Stephenson F.A. (2005) GRIF-1 and OIP106, members of a novel gene family of coiled-coil domain proteins: association in vivo and in vitro with kinesin. J. Biol. Chem., 280, 14723–14732. 15644324 10.1074/jbc.M409095200

[21] MacAskill A.F., Brickley K., Stephenson F.A., Kittler J.T. (2009) GTPase dependent recruitment of Grif-1 by Miro1 regulates mitochondrial trafficking in hippocampal neurons. Mol. Cell. Neurosci., 40, 301–312. 19103291 10.1016/j.mcn.2008.10.016

[22] van Spronsen M., Mikhaylova M., Lipka J., Schlager M.A., van den Heuvel D.J., Kuijpers M., Wulf P.S., Keijzer N., Demmers J., Kapitein L.C. (2013) TRAK/Milton motor-adaptor proteins steer mitochondrial trafficking to axons and dendrites. Neuron, 77, 485–502. 23395375 10.1016/j.neuron.2012.11.027

[23] Wang X., Winter D., Ashrafi G., Schlehe J., Wong Y.L., Selkoe D., Rice S., Steen J., LaVoie M.J., Schwarz T.L. (2011) PINK1 and Parkin target Miro for phosphorylation and degradation to arrest mitochondrial motility. Cell, 147, 893–906. 22078885 10.1016/j.cell.2011.10.018 PMC3261796

[24] Wang X., Schwarz T.L. (2009) The mechanism of Ca2+ -dependent regulation of kinesin-mediated mitochondrial motility. Cell, 136, 163–174. 19135897 10.1016/j.cell.2008.11.046 PMC2768392

[25] Macaskill A.F., Rinholm J.E., Twelvetrees A.E., Arancibia-Carcamo I.L., Muir J., Fransson A., Aspenstrom P., Attwell D., Kittler J.T. (2009) Miro1 is a calcium sensor for glutamate receptor-dependent localization of mitochondria at synapses. Neuron, 61, 541–555. 19249275 10.1016/j.neuron.2009.01.030 PMC2670979

[26] Weihofen A., Thomas K.J., Ostaszewski B.L., Cookson M.R., Selkoe D.J. (2009) Pink1 forms a multiprotein complex with Miro and Milton, linking Pink1 function to mitochondrial trafficking. Biochemistry, 48, 2045–2052. 19152501 10.1021/bi8019178 PMC2693257

[27] Shlevkov E., Kramer T., Schapansky J., LaVoie M.J., Schwarz T.L. (2016) Miro phosphorylation sites regulate Parkin recruitment and mitochondrial motility. Proc. Natl. Acad. Sci. USA, 113, E6097–E6106. 27679849 10.1073/pnas.1612283113 PMC5068282

[28] De Vos K.J., Sheetz M.P. (2007) Visualization and quantification of mitochondrial dynamics in living animal cells. Methods Cell Biol., 80, 627–682. 17445716 10.1016/S0091-679X(06)80030-0

[29] Mórotz G.M., De Vos K.J., Vagnoni A., Ackerley S., Shaw C.E., Miller C.C. (2012) Amyotrophic lateral sclerosis-associated mutant VAPBP56S perturbs calcium homeostasis to disrupt axonal transport of mitochondria. Hum. Mol. Genet, 21, 1979–1988. 22258555 10.1093/hmg/dds011 PMC3315205

[30] De Vos K.J., Mórotz G.M., Stoica R., Tudor E.L., Lau K.F., Ackerley S., Warley A., Shaw C.E., Miller C.C. (2012) VAPB interacts with the mitochondrial protein PTPIP51 to regulate calcium homeostasis. Hum. Mol. Genet., 21, 1299–1311. 22131369 10.1093/hmg/ddr559 PMC3284118

[31] Zhang F., Wang W., Siedlak S.L., Liu Y., Liu J., Jiang K., Perry G., Zhu X., Wang X. (2015) Miro1 deficiency in amyotrophic lateral sclerosis. Front. Aging Neurosci., 7, 100. 26074815 10.3389/fnagi.2015.00100 PMC4443026

[32] López-Doménech G., Higgs N.F., Vaccaro V., Roš H., Arancibia-Cárcamo I.L., MacAskill A.F., Kittler J.T. (2016) Loss of dendritic complexity precedes neurodegeneration in a mouse model with disrupted mitochondrial distribution in mature dendrites. Cell Rep., 17, 317–327. 27705781 10.1016/j.celrep.2016.09.004 PMC5067282

[33] Denison S.R., Wang F., Becker N.A., Schüle B., Kock N., Phillips L.A., Klein C., Smith D.I. (2003) Alterations in the common fragile site gene Parkin in ovarian and other cancers. Oncogene, 22, 8370–8378. 14614460 10.1038/sj.onc.1207072

[34] Pawlyk A.C., Giasson B.I., Sampathu D.M., Perez F.A., Lim K.L., Dawson V.L., Dawson T.M., Palmiter R.D., Trojanowski J.Q., Lee V.M. (2003) Novel monoclonal antibodies demonstrate biochemical variation of brain parkin with age. J. Biol. Chem., 278, 48120–48128. 12972409 10.1074/jbc.M306889200

[35] Narendra D., Tanaka A., Suen D.F., Youle R.J. (2008) Parkin is recruited selectively to impaired mitochondria and promotes their autophagy. J. Cell Biol., 183, 795–803. 19029340 10.1083/jcb.200809125 PMC2592826

[36] Magrané J., Cortez C., Gan W.B., Manfredi G. (2014) Abnormal mitochondrial transport and morphology are common pathological denominators in SOD1 and TDP43 ALS mouse models. Hum. Mol. Genet., 23, 1413–1424. 24154542 10.1093/hmg/ddt528 PMC3929084

[37] Magrané J., Hervias I., Henning M.S., Damiano M., Kawamata H., Manfredi G. (2009) Mutant SOD1 in neuronal mitochondria causes toxicity and mitochondrial dynamics abnormalities. Hum. Mol. Genet., 18, 4552–4564. 19779023 10.1093/hmg/ddp421 PMC2773270

[38] Eiyama A., Okamoto K. (2015) PINK1/Parkin-mediated mitophagy in mammalian cells. Curr. Opin. Cell Biol., 33, 95–101. 25697963 10.1016/j.ceb.2015.01.002

[39] Israelson A., Arbel N., Da Cruz S., Ilieva H., Yamanaka K., Shoshan-Barmatz V., Cleveland D.W. (2010) Misfolded mutant SOD1 directly inhibits VDAC1 conductance in a mouse model of inherited ALS. Neuron, 67, 575–587. 20797535 10.1016/j.neuron.2010.07.019 PMC2941987

[40] Higgins C.M.J., Jung C.W., Ding H.L., Xu Z.S. (2002) Mutant Cu, Zn superoxide dismutase that causes motoneuron degeneration is present in mitochondria in the CNS. J. Neurosci., 22, RC215. 11886899 10.1523/JNEUROSCI.22-06-j0001.2002 PMC6758252

[41] Ferri A., Cozzolino M., Crosio C., Nencini M., Casciati A., Gralla E.B., Rotilio G., Valentine J.S., Carrì M.T. (2006) Familial ALS-superoxide dismutases associate with mitochondria and shift their redox potentials. Proc. Natl. Acad. Sci. USA, 103, 13860–13865. 16945901 10.1073/pnas.0605814103 PMC1557633

[42] Mattiazzi M., D'Aurelio M., Gajewski C.D., Martushova K., Kiaei M., Beal M.F., Manfredi G. (2002) Mutated human SOD1 causes dysfunction of oxidative phosphorylation in mitochondria of transgenic mice. J. Biol. Chem., 277, 29626–29633. 12050154 10.1074/jbc.M203065200

[43] Pasinelli P., Belford M.E., Lennon N., Bacskai B.J., Hyman B.T., Trotti D., Brown R.H. (2004) Amyotrophic lateral sclerosis-associated SOD1 mutant proteins bind and aggregate with Bcl-2 in spinal cord mitochondria. Neuron, 43, 19–30. 15233914 10.1016/j.neuron.2004.06.021

[44] Vande Velde C., Miller T.M., Cashman N.R., Cleveland D.W. (2008) Selective association of misfolded ALS-linked mutant SOD1 with the cytoplasmic face of mitochondria. Proc. Natl. Acad. Sci. USA, 105, 4022–4027. 18296640 10.1073/pnas.0712209105 PMC2268797

[45] Ahtoniemi T., Jaronen M., Keksa-Goldsteine V., Goldsteins G., Koistinaho J. (2008) Mutant SOD1 from spinal cord of G93A rats is destabilized and binds to inner mitochondrial membrane. Neurobiol. Dis., 32, 479–485. 18817872 10.1016/j.nbd.2008.08.010

[46] Lautenschlager J., Prell T., Ruhmer J., Weidemann L., Witte O.W., Grosskreutz J. (2013) Overexpression of human mutated G93A SOD1 changes dynamics of the ER mitochondria calcium cycle specifically in mouse embryonic motor neurons. Exp. Neurol., 247, 91–100. 23578819 10.1016/j.expneurol.2013.03.027

[47] Guatteo E., Carunchio I., Pieri M., Albo F., Canu N., Mercuri N.B., Zona C. (2007) Altered calcium homeostasis in motor neurons following AMPA receptor but not voltage-dependent calcium channels’ activation in a genetic model of amyotrophic lateral sclerosis. Neurobiol. Dis., 28, 90–100. 17706428 10.1016/j.nbd.2007.07.002

[48] Kruman I.I., Pedersen W.A., Springer J.E., Mattson M.P. (1999) ALS-linked Cu/Zn-SOD mutation increases vulnerability of motor neurons to excitotoxicity by a mechanism involving increased oxidative stress and perturbed calcium homeostasis. Exp. Neurol., 160, 28–39. 10630188 10.1006/exnr.1999.7190

[49] Irvin C.W., Kim R.B., Mitchell C.S. (2015) Seeking homeostasis: temporal trends in respiration, oxidation, and calcium in SOD1 G93A Amyotrophic Lateral Sclerosis mice. Front. Cell. Neurosci., 9, 248. 26190973 10.3389/fncel.2015.00248 PMC4486844

[50] Wang W., Li L., Lin W.L., Dickson D.W., Petrucelli L., Zhang T., Wang X. (2013) The ALS disease-associated mutant TDP-43 impairs mitochondrial dynamics and function in motor neurons. Hum. Mol. Genet., 22, 4706–4719. 23827948 10.1093/hmg/ddt319 PMC3820133

[51] Baldwin K.R., Godena V.K., Hewitt V.L., Whitworth A.J. (2016) Axonal transport defects are a common phenotype in Drosophila models of ALS. Hum. Mol. Genet., 25, 2378–2392. 27056981 10.1093/hmg/ddw105 PMC5181624

[52] Chen Y., Deng J., Wang P., Yang M., Chen X., Zhu L., Liu J., Lu B., Shen Y., Fushimi K., Xu Q., Wu J.Y. (2016) PINK1 and Parkin are genetic modifiers for FUS-induced neurodegeneration. Hum. Mol. Genet., 25, 5059–5068. 27794540 10.1093/hmg/ddw310 PMC6078632

[53] Bernard-Marissal N., Médard J.J., Azzedine H., Chrast R. (2015) Dysfunction in endoplasmic reticulum-mitochondria crosstalk underlies SIGMAR1 loss of function mediated motor neuron degeneration. Brain, 138, 875–890. 25678561 10.1093/brain/awv008

[54] Sasaki S., Iwata M. (2007) Mitochondrial alterations in the spinal cord of patients with sporadic amyotrophic lateral sclerosis. J. Neuropathol. Exp. Neurol., 66, 10–16. 17204932 10.1097/nen.0b013e31802c396b

[55] Sasaki S., Iwata M. (1996) Ultrastructural study of synapses in the anterior horn neurons of patients with amyotrophic lateral sclerosis. Neurosci. Lett., 204, 53–56. 8929976 10.1016/0304-3940(96)12314-4

[56] Hirano A., Nakano I., Kurland L.T., Mulder D.W., Holley P.W., Saccomanno G. (1984) Fine structural study of neurofibrillary changes in a family with amyotrophic lateral sclerosis. J. Neuropathol. Exp. Neurol., 43, 471–480. 6540800 10.1097/00005072-198409000-00002

[57] Grosskreutz J., Van Den Bosch L., Keller B.U. (2010) Calcium dysregulation in amyotrophic lateral sclerosis. Cell Calcium, 47, 165–174. 20116097 10.1016/j.ceca.2009.12.002

[58] Deng J., Yang M., Chen Y., Chen X., Liu J., Sun S., Cheng H., Li Y., Bigio E.H., Mesulam M. (2015) FUS interacts with HSP60 to promote mitochondrial damage. PLoS Genet., 11, e1005357. 26335776 10.1371/journal.pgen.1005357 PMC4559378

[59] Wang W., Wang L., Lu J., Siedlak S.L., Fujioka H., Liang J., Jiang S., Ma X., Jiang Z., da Rocha E.L. (2016) The inhibition of TDP-43 mitochondrial localization blocks its neuronal toxicity. Nat. Med., 22, 869–878. 27348499 10.1038/nm.4130 PMC4974139

[60] Lopez-Gonzalez R., Lu Y., Gendron T.F., Karydas A., Tran H., Yang D., Petrucelli L., Miller B.L., Almeida S., Gao F.B. (2016) Poly(GR) in C9ORF72-related ALS/FTD compromises mitochondrial function and increases oxidative stress and DNA damage in iPSC-derived motor neurons. *Neuron*, 92, 383–391. 10.1016/j.neuron.2016.09.015 PMC5111366 27720481

[61] Mutihac R., Alegre-Abarrategui J., Gordon D., Farrimond L., Yamasaki-Mann M., Talbot K., Wade-Martins R. (2015) TARDBP pathogenic mutations increase cytoplasmic translocation of TDP-43 and cause reduction of endoplasmic reticulum Ca^2+^ signaling in motor neurons. Neurobiol. Dis., 75, 64–77. 25526708 10.1016/j.nbd.2014.12.010

[62] Stoica R., Paillusson S., Gomez-Suaga P., Mitchell J.C., Lau D.H., Gray E.H., Sancho R.M., Vizcay-Barrena G., De Vos K.J., Shaw C.E. (2016) ALS/FTD-associated FUS activates GSK-3β to disrupt the VAPB-PTPIP51 interaction and ER-mitochondria associations. EMBO Rep., 17, 1326–1342. 27418313 10.15252/embr.201541726 PMC5007559

[63] Stoica R., De Vos K.J., Paillusson S., Mueller S., Sancho R.M., Lau K.F., Vizcay-Barrena G., Lin W.L., Xu Y.F., Lewis J. (2014) ER-mitochondria associations are regulated by the VAPB-PTPIP51 interaction and are disrupted by ALS/FTD-associated TDP-43. Nat. Commun., 5, 3996. 24893131 10.1038/ncomms4996 PMC4046113

[64] Watanabe S., Ilieva H., Tamada H., Nomura H., Komine O., Endo F., Jin S., Mancias P., Kiyama H., Yamanaka K. (2016) Mitochondria-associated membrane collapse is a common pathomechanism in SIGMAR1- and SOD1-linked ALS. EMBO Mol. Med., 8, 1421–1437. 27821430 10.15252/emmm.201606403 PMC5167132

[65] Gregianin E., Pallafacchina G., Zanin S., Crippa V., Rusmini P., Poletti A., Fang M., Li Z., Diano L., Petrucci A. (2016) Loss-of-function mutations in the SIGMAR1 gene cause distal hereditary motor neuropathy by impairing ER-mitochondria tethering and Ca2+ signalling. Hum. Mol. Genet., 25, 3741–3753. 27402882 10.1093/hmg/ddw220

[66] Siklós L., Engelhardt J., Harati Y., Smith R.G., Joó F., Appel S.H. (1996) Ultrastructural evidence for altered calcium in motor nerve terminals in amyotropic lateral sclerosis. Ann. Neurol., 39, 203–216. 8967752 10.1002/ana.410390210

[67] Zhu Y.B., Sheng Z.H. (2011) Increased axonal mitochondrial mobility does not slow amyotrophic lateral sclerosis (ALS)-like disease in mutant SOD1 mice. J. Biol. Chem., 286, 23432–23440. 21518771 10.1074/jbc.M111.237818 PMC3123107

[68] Stewart S.A., Dykxhoorn D.M., Palliser D., Mizuno H., Yu E.Y., An D.S., Sabatini D.M., Chen I.S., Hahn W.C., Sharp P.A. (2003) Lentivirus-delivered stable gene silencing by RNAi in primary cells. RNA, 9, 493–501. 12649500 10.1261/rna.2192803 PMC1370415

[69] Dull T., Zufferey R., Kelly M., Mandel R.J., Nguyen M., Trono D., Naldini L. (1998) A third-generation lentivirus vector with a conditional packaging system. J. Virol., 72, 8463–8471. 9765382 10.1128/jvi.72.11.8463-8471.1998 PMC110254

[70] Webster C.P., Smith E.F., Bauer C.S., Moller A., Hautbergue G.M., Ferraiuolo L., Myszczynska M.A., Higginbottom A., Walsh M.J., Whitworth A.J. (2016) The C9orf72 protein interacts with Rab1a and the ULK1 complex to regulate initiation of autophagy. EMBO J., 35, 1656–1676. 27334615 10.15252/embj.201694401 PMC4969571

[71] Henderson C., Bloch-Gallego E., Camu W. (1995) Purified embryonic motoneurons In Cohen J., Wilkin G. (eds), Nerve Cell culture: A Practical Approach. Oxford University Press, London, UK, pp. 69–81.

[72] Schindelin J., Arganda-Carreras I., Frise E., Kaynig V., Longair M., Pietzsch T., Preibisch S., Rueden C., Saalfeld S., Schmid B. (2012) Fiji: an open-source platform for biological-image analysis. Nat. Methods, 9, 676–682. 22743772 10.1038/nmeth.2019 PMC3855844

[73] Schneider C.A., Rasband W.S., Eliceiri K.W. (2012) NIH Image to ImageJ: 25 years of image analysis. Nat. Methods, 9, 671–675. 22930834 10.1038/nmeth.2089 PMC5554542

[74] Edelstein A.D., Tsuchida M.A., Amodaj N., Pinkard H., Vale R.D., Stuurman N. (2014) Advanced methods of microscope control using μManager software. J. Biol. Methods, 1, e10. 25606571 10.14440/jbm.2014.36 PMC4297649

