# Supplementary figures and images for: Amyotrophic lateral sclerosis-associated mutant SOD1 inhibits anterograde axonal transport of mitochondria by reducing Miro1 levels
Source: Hum Mol Genet. 2017 Sep 14;26(23):4668–79. doi: 10.1093/hmg/ddx348 (PMC5886184; doi:10.1093/hmg/ddx348)

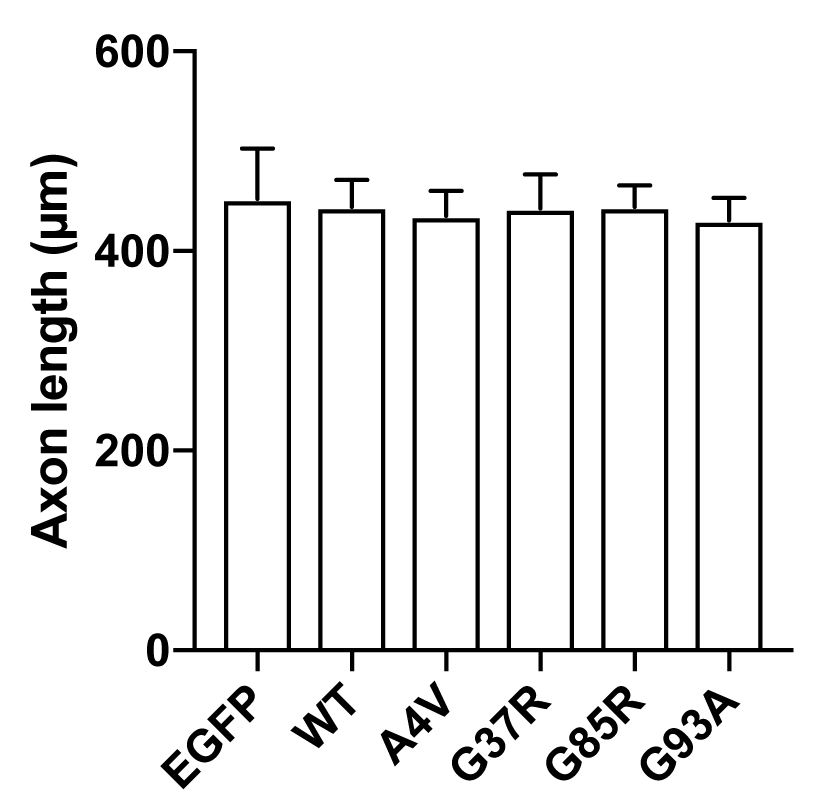

Supplement: Supplementary Figure S1 [file sfig1_ddx348.png]

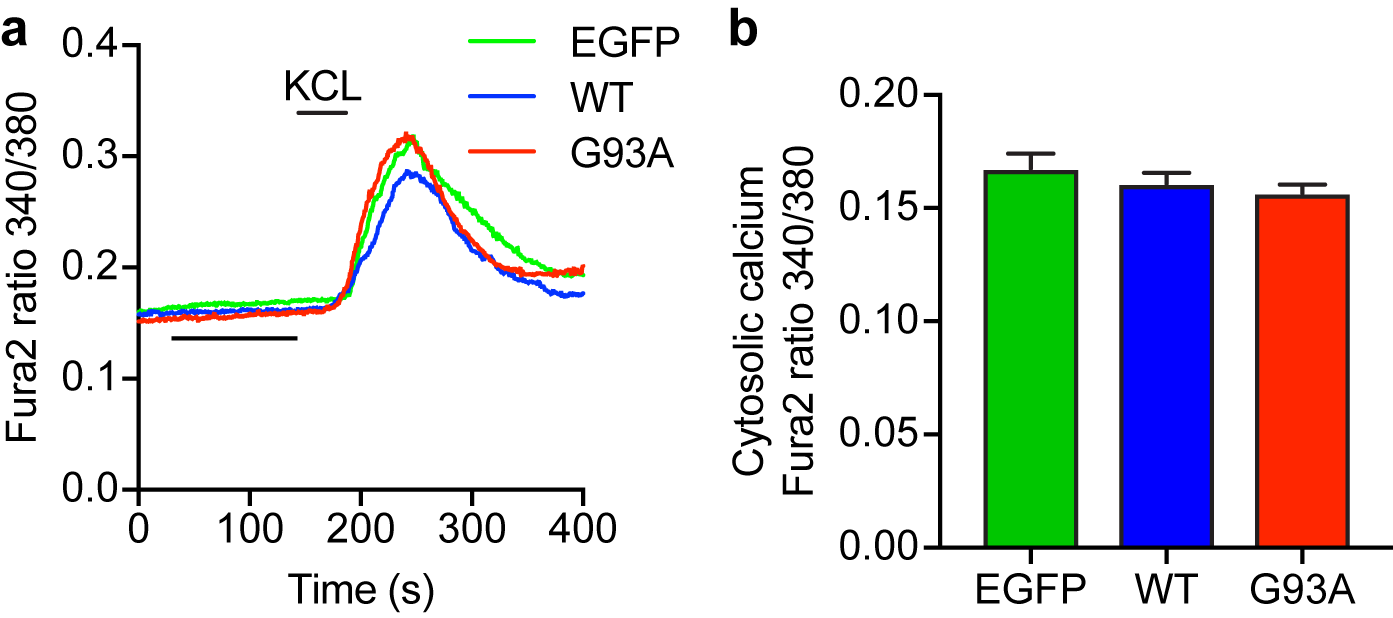

Supplement: Supplementary Figure S2 [file sfig2_ddx348.png]

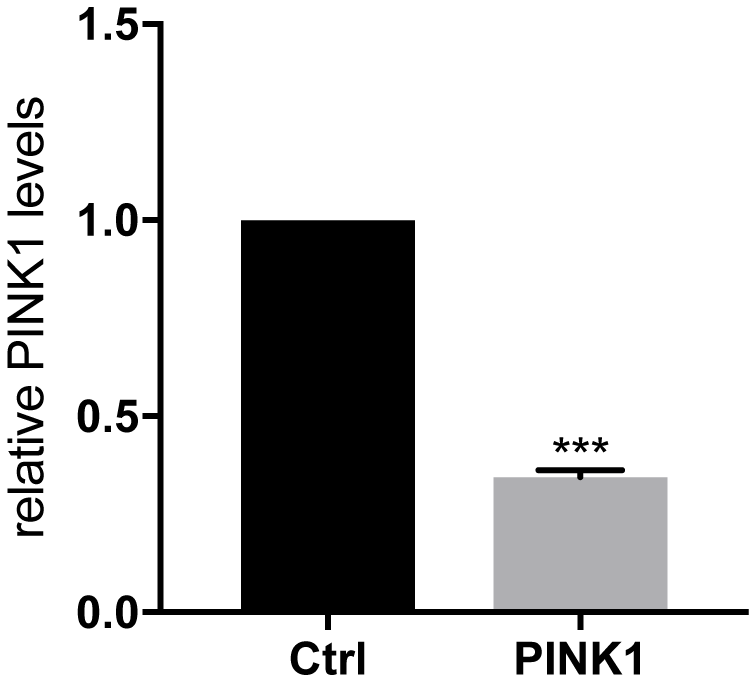

Supplement: Supplementary Figure S3 [file sfig3_ddx348.png]
